# A new species of *Lysiteles* Simon, 1895 (Araneae, Thomisidae) from South China

**DOI:** 10.3897/BDJ.10.e95981

**Published:** 2022-12-19

**Authors:** Wenhui Li, Congzheng Li, Yanbin Yao, Keke Liu, Xuqing Liao

**Affiliations:** 1 Jinggangshan University, Ji'an, China Jinggangshan University Ji'an China; 2 Jinshan College of Fujian Agriculture And Forestry University, Fuzhou, China Jinshan College of Fujian Agriculture And Forestry University Fuzhou China; 3 Nanfengmian National Nature Reserve Administration Centre, Ji'an, China Nanfengmian National Nature Reserve Administration Centre Ji'an China

**Keywords:** axonomy, spiders, morphological, Jiangxi

## Abstract

**Background:**

*Lysiteles* Simon, 1895 is one of the largest taxa with small body size among the Thomisidae and is mainly distributed in East, South and Southeast Asia. Most of them are recorded from southern provinces of China, such as Jiangxi Province, including three species. However, all of them are only discovered from Jinggang Mountain National Nature Reserve in Jiangxi Province. But there are still other species remaining unknown which need to be surveyed from other areas in this Province.

**New information:**

One *Lysiteles* species was collected from Nanfengmian National Nature Reserve in Jiangxi Province. Based on morphological characters, it was recognised as a new species and has been named as *Lysitelesnanfengmian* **sp. n.** It is described and illustrated with photographs and its distribution is also mapped.

## Introduction

The species of the genus *Lysiteles* Simon, 1895 usually live in shrubs, grasses, tree foliage and leaf litter, sometimes in canopy (Tang et al. 2008 and our experience). Tang et al. (2007) and Tang et al. (2008) characterised the genus by the combination of the diverse conspicuous blackish-brown markings on the dorsal body, the indistinct cervical groove, radial grooves and fovea, the palpal tegulum lacking tegular apophysis (in most species) and the round or oval separated spermathecae on female epigynes. Currently, there are 61 nominal species within the genus, but more than 25 species known by one sex only including 16 species from China (World Spider Catalog 2022). Additionally, 16 species of them were recorded from China alone (World Spider Catalog 2022). So currently taxonomic work of the genus becomes difficult to many arachnologists in this country.

The genus has a relatively wide distribution in East, South and Southeast Asia, with thehighest number species in China (44), Bhutan (ten) and Nepal (eight) (World Spider Catalog 2022). Other countries have fewer than six, such as Philippines (five), Japan (four), India (three) and the Far East of Russia (two) (World Spider Catalog 2022). Nearly 1/3 of them were recorded from Yunnan Province (Tang et al. 2008). Thanks to the study of Tang et al. who described 25 species from Yunnan Province (Tang et al. 2008), we now know that these spiders are very common in shrubs and leaf litter. Meanwhile, it is further remarkable that most of the descriptions are based on a few specimens of a single sex (Tang et al. 2008). This is mainly due to the fact that *Lysiteles* species have small body size and were almost exclusively collected in shrubs and leaf litter.

From 2015 to 2022, many spider taxa have been discovered by our team in south-central Jiangxi Province, such as Agelenidae (Liu et al. 2020a, Liu et al. 2021), Dictynidae (Liu et al. 2018), Oonopidae (Liu et al. 2016, Liu et al. 2019), Phrurolithidae (Liu et al. 2020b, Liu et al. 2020c, Liu et al. 2021, Liu et al. 2022d), Salticidae (Liu et al. 2017b, Ying et al. 2021, Liu et al. 2022a), Thomisidae (Liu et al. 2017a, Liu et al. 2022c) and Gnaphosidae (Liu et al. 2022b). Previous research recorded more than 100 new species in this area and most of them are endemic to this country. These discoveries support the contention that Jiangxi Province is one of China's biodiversity hotspots. These results also confirm that most other regions are still underexplored and that detailed and systematic research is needed.

Jiangxi Nanfengmian National Nature Reserve is located in the south-central section of Luoxiao Mountains and on the common boundary of Jiangxi and Hunan Provinces, which ha a subtropical monsoon climate. While studying thomisid spiders from there, one undescribed and poorly known species was found. The aim of the present paper is to provide a detailed description of this new species.

## Materials and methods

Specimens were examined using a SZ6100 stereomicroscope. Both male and female copulatory organs were dissected and examined in 80% ethanol using an Olympus CX43 compound microscope with a KUY NICE CCD camera. Epigynes were cleared with pancreatin solution (Álvarez-Padilla and Hormiga 2007). Specimens, including dissected male palps and epigynes, were preserved in 75% ethanol after examination. For scanning electron micrographs (SEMs), specimens were dried under natural conditions, sprayed with gold with a small ion-sputtering apparatus ETD-2000 or left without coating and photographed with a ZEISS EVO LS15 scanning electron microscope. All specimens including the holotype are deposited in the Animal Specimen Museum, College of Life Science, Jinggangshan University (ASM-JGSU).

The measurements were taken using a stereomicroscope (AxioVision SE64 Rel. 4.8.3) and are given in millimetres. The body lengths of all specimens exclude the chelicerae and spinnerets. Terminology of the male and female genitalia follows Benjamin (2011) and Liu et al. (2022c).

Leg measurements are given as total length (femur, patella, tibia, metatarsus, tarsus). The abbreviations used in the figures and text are as follows: ALE − anterior lateral eye, AME − anterior median eye, At − atrium, CD − copulatory ducts, CO − copulatory openings, d − dorsal, Em − embolus, Fe − femur, MOA − median ocular area, p − prolateral, Pa − patella, PLE − posterior lateral eye, PME − posterior median eye, r − retrolateral, RTA − retrolateral tibial apophysis, Se − septum, Spe − spermathecae, Ti − tibia, TR − Tegular ridge, v − ventral, VTA − ventral tibial apophysis.

## Taxon treatments

### Lysiteles nanfengmian

Liu sp. n.

5E97CDC1-B222-5EFE-B252-C5651C417D0B

EE0E97F7-088A-40E9-AA49-04F9C9E8F798

#### Materials

- **Type status:** Holotype. **Occurrence:** recordedBy: Liu Ke-Ke; individualCount: 1; sex: male; lifeStage: adult; occurrenceID: FB98876F-2A09-520E-A579-173D2584BA7B; **Taxon:** scientificName: *Lysitelesnanfengmian* Liu, sp. n.; **Location:** country: China; stateProvince: Jiangxi; locality: Ji’an City, Suichuan County, Nanfengmian National Nature Reserve, Daijiabu Station, Shahu Village, Fengshuao; verbatimElevation: 1071 m; verbatimCoordinates: 26°16'1.33"N, 114°3'47.53"E; georeferenceProtocol: GPS; **Event:** samplingProtocol: sweeping; eventDate: 28/06/2022
- **Type status:** Paratype. **Occurrence:** recordedBy: Liu Ke-Ke; individualCount: 1; sex: male; lifeStage: adult; occurrenceID: BB9DAF8E-5DD6-5AB4-BD63-90663760BE52; **Taxon:** scientificName: *Lysitelesnanfengmian* Liu, sp. n.; **Location:** country: China; stateProvince: Jiangxi; locality: Ji’an City, Suichuan County, Nanfengmian National Nature Reserve, Daijiabu Station, Shahu Village, Fengshuao; verbatimElevation: 1071 m; verbatimCoordinates: 26°16'1.33"N, 114°3'47.53"E; georeferenceProtocol: GPS; **Event:** samplingProtocol: sweeping; eventDate: 28/06/2022
- **Type status:** Paratype. **Occurrence:** recordedBy: Liu Ke-Ke; individualCount: 4; sex: female; lifeStage: adult; occurrenceID: B716A5F2-00A8-533A-A8BA-962217465B8B; **Taxon:** scientificName: *Lysitelesnanfengmian* Liu, sp. n.; **Location:** country: China; stateProvince: Jiangxi; locality: Ji’an City, Suichuan County, Nanfengmian National Nature Reserve, Daijiabu Station, Shahu Village, Fengshuao; verbatimElevation: 1071 m; verbatimCoordinates: 26°16'1.33"N, 114°3'47.53"E; georeferenceProtocol: GPS; **Event:** samplingProtocol: sweeping; eventDate: 28/06/2022
- **Type status:** Paratype. **Occurrence:** recordedBy: Liu Ke-Ke; individualCount: 4; sex: female; lifeStage: adult; occurrenceID: 5EA24413-7755-5D56-9503-D509339C7DD4; **Taxon:** scientificName: *Lysitelesnanfengmian* Liu, sp. n.; **Location:** country: China; stateProvince: Jiangxi; locality: Ji’an City, Suichuan County, Nanfengmian National Nature Reserve, Daijiabu Station, Qianmo Village, Dapingli; verbatimElevation: 1096 m; verbatimCoordinates: 26°17'44.2"N, 114°4'29.74"E; georeferenceProtocol: GPS; **Event:** samplingProtocol: sweeping; eventDate: 28/06/2022
- **Type status:** Paratype. **Occurrence:** recordedBy: Liu Ke-Ke; individualCount: 1; sex: male; lifeStage: adult; occurrenceID: 43300CF0-FE44-5008-836C-337E5D52EC3B; **Taxon:** scientificName: *Lysitelesnanfengmian* Liu, sp. n.; **Location:** country: China; stateProvince: Jiangxi; locality: Ji’an City, Suichuan County, Nanfengmian National Nature Reserve, Dafen Station, Gaoxing Village, Shiziao; verbatimElevation: 913 m; verbatimCoordinates: 26°20'28.85"N, 114°5'27.47"E; georeferenceProtocol: GPS; **Event:** samplingProtocol: sweeping; eventDate: 27/06/2022
- **Type status:** Paratype. **Occurrence:** recordedBy: Liu Ke-Ke; individualCount: 1; sex: female; lifeStage: adult; occurrenceID: 0D27B20F-5394-57C3-A7E4-11E35C95D4FE; **Taxon:** scientificName: *Lysitelesnanfengmian* Liu, sp. n.; **Location:** country: China; stateProvince: Jiangxi; locality: Ji’an City, Suichuan County, Nanfengmian National Nature Reserve, Dafen Station, Gaoxing Village, Shiziao; verbatimElevation: 913 m; verbatimCoordinates: 26°20'28.85"N, 114°5'27.47"E; georeferenceProtocol: GPS; **Event:** samplingProtocol: sweeping; eventDate: 27/06/2022

#### Description

**Figure 1.**
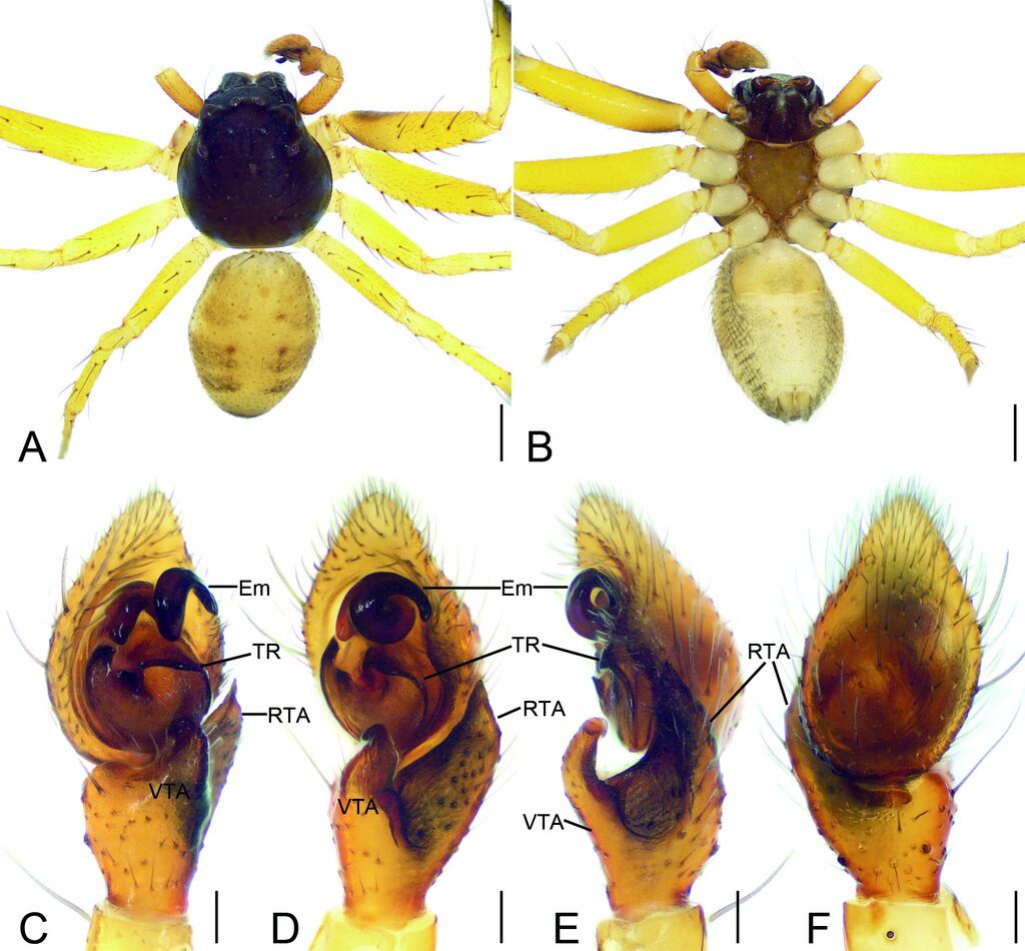
*Lysitelesnanfengmian* sp. n., male holotype. **A** habitus, dorsal view; **B** same, ventral view; **C** palp, prolatero-ventral view; **D** same, ventral view; **E** same, retrolatero-dorsal view; **F** same, dorsal view. Abbreviations: Em − embolus, RTA − retrolateral tibial apophysis, TR − Tegular ridge, VTA − ventral tibial apophysis. Scale bars: 0.2 mm (**A, B**), 0.1 mm (**C–F**).

**Male** (holotype) (Figs 1, 4A, B). Total length 3.42. Carapace (Fig. 1A, B) 1.68 long, 1.59 wide. Eye sizes and interdistances: AME 0.10, ALE 0.20, PME 0.07, PLE 0.15, AME−AME 0.15, AME−ALE 0.16, PME−PME 0.25, PME−PLE 0.27, AME−PME 0.26, AME−PLE 0.50, ALE−ALE 0.65, PLE−PLE 0.80, ALE−PLE 0.24. MOA 0.30 long, front width 0.33, back width 0.41. Chelicerae without promaiginal tooth, but with two retromarginal teeth. Endites two times as long as the maximum width, anteriorly with dense setae, medially with distinct constriction. Labium wider than two times length, anteriorly with four to six strong setae, subposteriorly with a constriction. Sternum shield-shaped, as long as wide, anteromedially with a shallow notch, laterally with serrulate and thickened margin, posterior end blunt. Legs (Fig. 1A, B): measurements: I 6.15 (1.79, 0.67, 1.61, 1.37, 0.71); II 6.65 (2.04, 0.58, 1.78, 1.45, 0.80); III 3.75 (1.11, 0.50, 1.00, 0.72, 0.42); IV 3.98 (1.08, 0.48, 1.06, 0.82, 0.54); formula: 2143; spination: I Fe: d5, p3, r1; Pa: d2, p1, r1; Ti: d2, p3, r3, v2; Mt: p2, r2, v3; II Fe: d6, r1; Pa: d3, r1; Ti: d6, p2, r2, v2; Mt: d4, p3, r3, v2; III Fe: r4; Pa: r2; Ti: d2, p2, r3, v1; Mt: d3, p3, v1; IV Fe: d4, r1; Pa: d3, r1; Ti: d4, p2, r2, v1; Mt: d3, p3, r3, v1. Abdomen (Fig. 1A, B) 1.71 long, 1.29 wide, dorsally with five round sigilla and abundant long setae; venter with five to six pairs of sigilla postero-medially.

Colouration (Fig. 1A and B). Carapace, chelicerae, endites and labium black brown. Sternum dark brown, lateral sub-margins with black brown stripes. Legs yellow, femora I and II with narrow prolateral and retrolateral stripes. Abdomen yellow, with four pairs of mottled spots dorsolaterally and the indistinct black-brown stripe in front of anal tubercle.

Palp (Fig. 1C−F and Fig. 3). Tibia with two apophyses: ventral apophysis (VTA) hook-shaped in ventral view, shorter than tibia, curved prolaterally, with widened base; retrolateral apophysis (RTA) thick, horn-like, with tooth-like apex, longer than tibia. Tegular ridge (TR) broad, arising at 12 o’clock position, extending along tegular margin. Embolus (Em) very thick, with 1.5 spirals clockwise, apex directed at 3 o’clock position.

**Figure 2.**
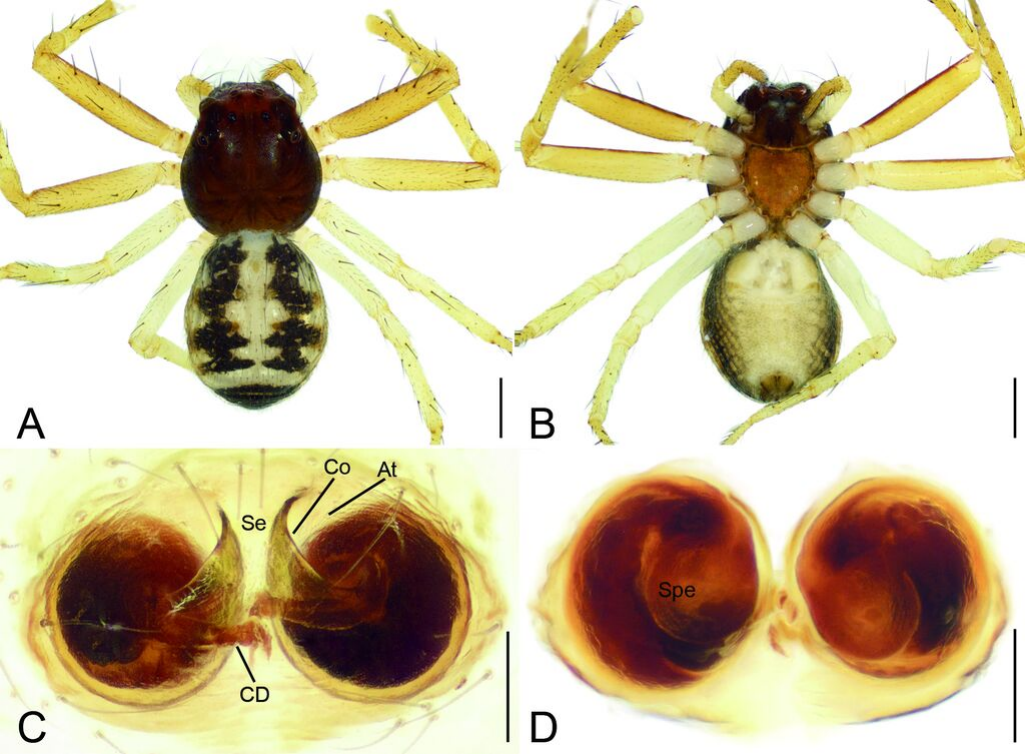
*Lysitelesnanfengmian* sp. n., female paratype. **A** habitus, dorsal view; **B** same, ventral view; **C** epigyne, ventral view; **D** vulva, dorsal view. Abbreviations: At − atrium, CD − copulatory ducts, CO − copulatory openings, Se − septum, Spe − spermathecae. Scale bars: 0.6 mm (**A, B**), 0.1 mm (**C–F**).

**Female** (Fig. 2 and Fig. 4C and D). As in male, except as noted. Total length 4.28. Carapace (Fig. 2A and B) 2.07 long, 2.02 wide. Eye sizes and interdistances: AME 0.11, ALE 0.20, PME 0.07, PLE 0.13, AME−AME 0.17, AME−ALE 0.14, PME−PME 0.27, PME−PLE 0.29, AME−PME 0.20, AME−PLE 0.49, ALE−ALE 0.64, PLE−PLE 0.83, ALE−PLE 0.25. MOA 0.27 long, front width 0.35, back width 0.41. Chelicerae without teeth. Leg measurements (Fig. 2A and B): I 4.89 (1.44, 0.60, 1.21, 0.99, 0.65); II 5.34 (1.64, 0.61, 1.34, 1.14, 0.61); III 3.15 (0.99, 0.44, 0.76, 0.58, 0.38); IV 3.70 (1.12, 0.51, 0.89, 0.71, 0.47); spination: I Fe: d3, p2; Pa: d1, p1; Ti: d2, p1, r4, v2; Mt: p2, r3, v2; II Fe: d3, r1; Pa: d2; Ti: d2, p3, r2, v3; Mt: d1, r1, v6; III Fe: d1; Pa: d2; Ti: d2, p1, r1, v1; Mt: d2, p2, r1, v3; IV Ti: v1; Mt: p1. Abdomen (Fig. 2C and D) 2.21 long, 1.96 wide.

Colouration (Fig. 2A and B). Carapace red to dark brown. Chelicerae, endites and labium dark brown. Sternum yellow brown, mottled. Abdomen with a few white spots, distinct symmetrical black-brown stripes from anterolaterally to subposterolaterally and the subtriangular black-brown spot in front of anus.

**Figure 3.**
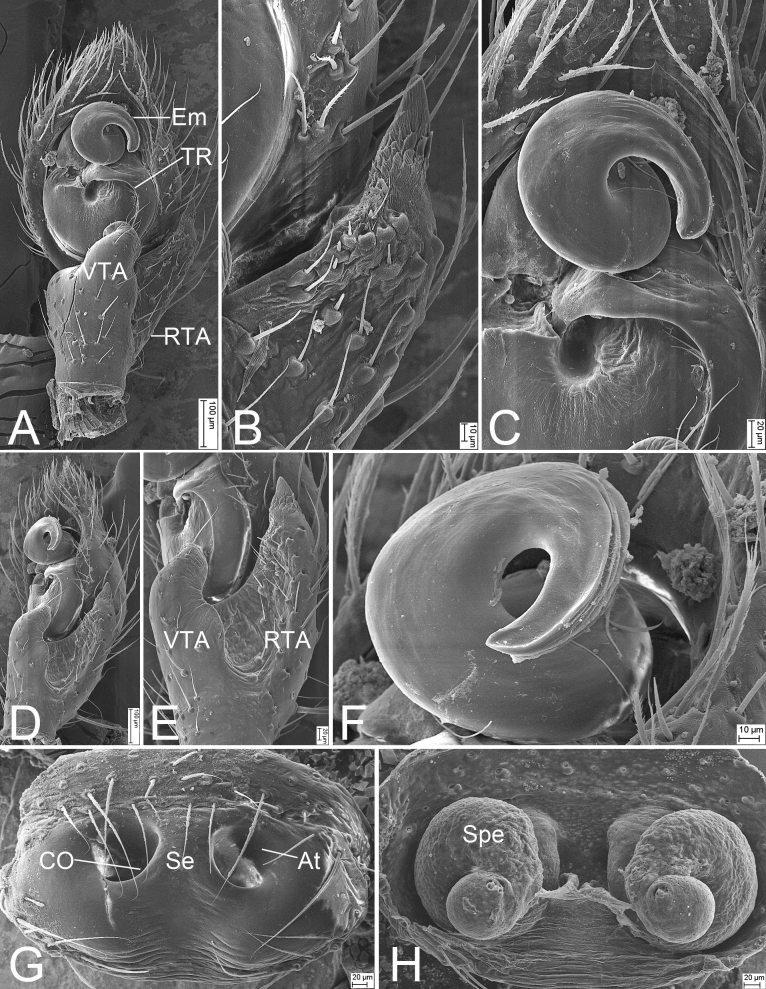
SEMs of *Lysitelesnanfengmian* sp. n., male palp and female epigyne, paratype. **A** palp, ventral view; **B** same, detail of RTA; **C** same, detail of Em and TR; **D** same, retro-ventral view; **E** same, detail of VTA and RTA; **F** same, detail of Em; **G** epigyne, ventral view; **H** vulva, dorsal view. Abbreviations: At − atrium, CD − copulatory ducts, CO − copulatory openings, Em − embolus, RTA − retrolateral tibial apophysis, Se − septum, Spe − spermathecae, TR − Tegular ridge, VTA − ventral tibial apophysis.

Epigyne (Fig. 2C and D and Fig. 3G and H) two times wider than long. Anteromedian part with septum (Se) dividing atrium (At) into two large oval parts. Copulatory openings (CO) located at posterior part of the fovea. Copulatory ducts (CD) almost straight, not in a line, shorter than spermathecal width. Spermathecae (Spe) anticlockwise coiled, forming one full turn, slightly separated by nearly 1/3 of its maximum width (in some specimens). Fertilisation duct indistinct.

**Figure 4.**
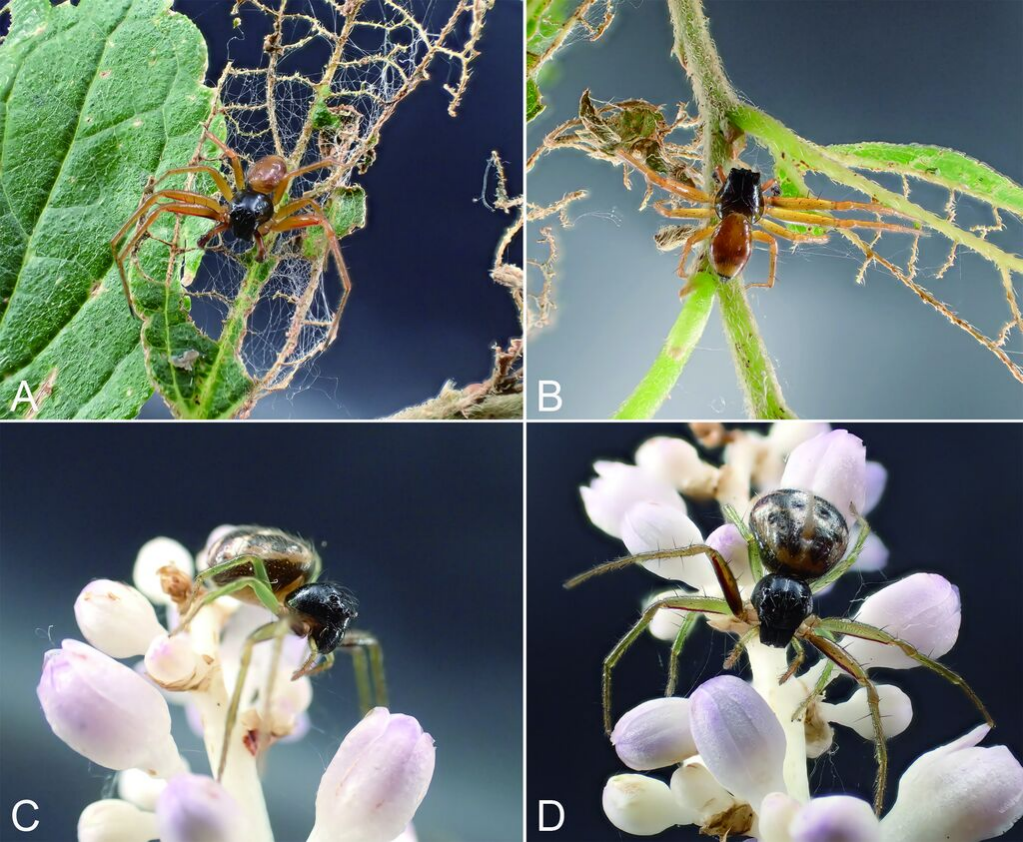
*Lysitelesnanfengmian* sp. n., living specimen. **A & B** male; **C & D** female.

Comments. In life (Fig. 4), this species exhibits dark colours in most parts of the legs and abdomen, while yellowish after preservation in ethanol.

#### Diagnosis

The male of this new species is similar to that of *Lysitelestorsivus* Zhang, Zhu & Tso, 2006 (Tang et al. 2008: 34, fig. 18a−e) in having a hook-shaped ventral tibial apophysis and thick cone-shaped retrolateral apophysis, but can be easily distinguished from it by (Fig. 1) the carapace lacking pair of broad yellow-brown stripes (vs present in *L.torsivus*) and the anterior part of embolus with 1.5 spirals (vs 1.25 in *L.torsivus*). The female of the new species resembles those of *L.auriculatus* Tang, Yin, Peng, Ubick & Griswold, 2008 (Tang et al. 2008: 6, fig. 2a−e), *L.spirellus* Tang, Yin, Peng, Ubick & Griswold, 2008 (Tang et al. 2008: 31, fig. 16a−f), *L.subspirellus* Liu, 2022 (Liu et al. 2022c: 55, fig. 7A, C, D) in having anticlockwise spiral spermathecae, but can be separated by (Fig. 2A, C and D) the longitudinal dark brown stripes covered 1/3 of dorsal abdomen (vs nearly 1/2 in *L.auriculatus* and *L.spirellus*, more than 2/3 in *L.subspirellus*) and the spermathecal end directed at 7 o’clock (vs 5 o’clock in *L.auriculatus*, 9 o’clock in *L.spirellus*, 6 o’clock in *L.subspirellus*).

#### Etymology

The specific name refers to the type locality; noun in apposition.

#### Distribution

**Figure 5.**
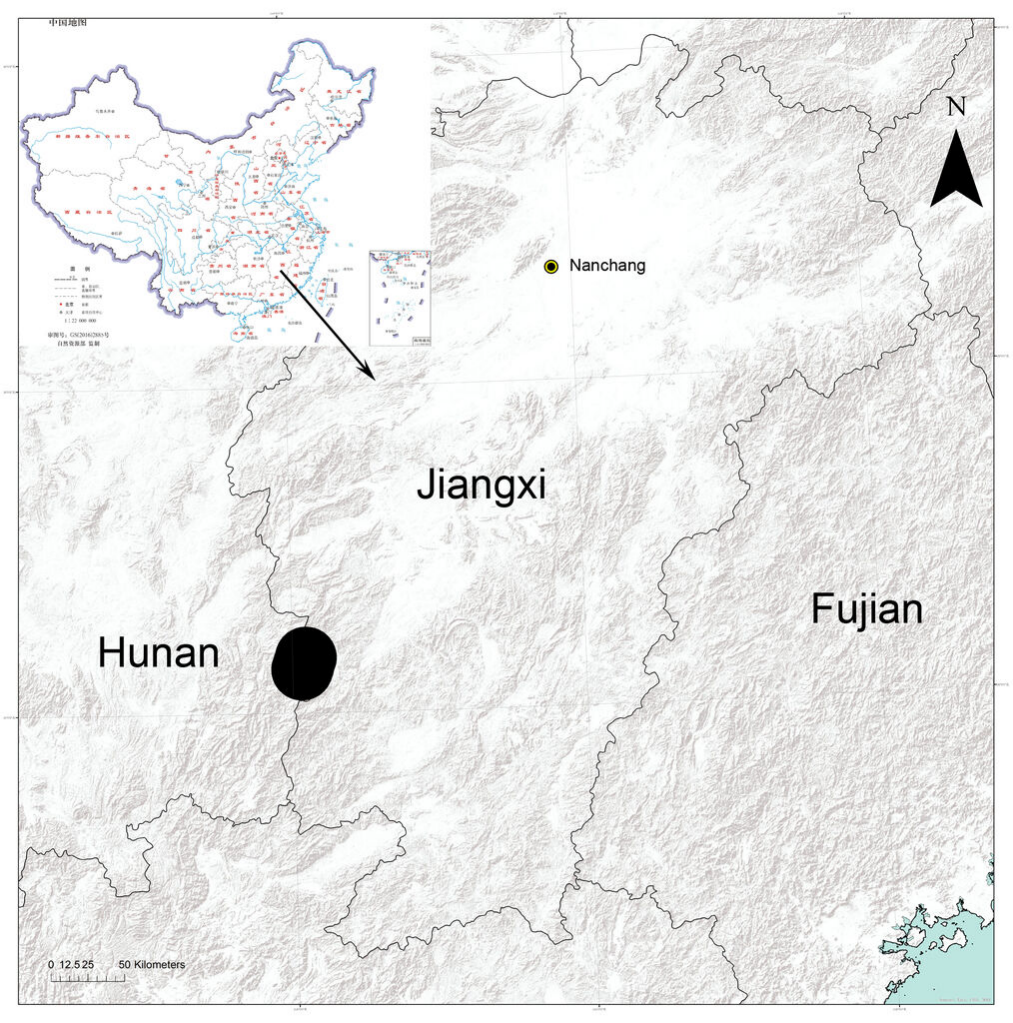
Records of *Lysitelesnanfengmian* sp. n. from Nanfengmian National Nature Reserve in Jiangxi Province, China.

Known only from the type locality in Jiangxi Province, China (Fig. 5).

## Discussion

When we collected all illustrated descriptions of the *Lysiteles* species, one of the interesting findings is that some of them can be assigned in one sub-group by the embolus with a spiral or strongly curved tip and the large RTA longer than the tibia and the female epigyne with the distinct septum dividing the atrium into two large parts, including *L.arcuatus* Tang, Yin, Peng, Ubick & Griswold, 2008, *L.auriculatus*, *L.corrugus* Tang, Yin, Peng, Ubick & Griswold, 2008, *L.dentatus* Tang, Yin, Peng, Ubick & Griswold, 2007, *L.dianicus* Song & Zhao, 1994, *L.qiuae* Song & Wang, 1991, *L.silvanus* Ono, 1980, *L.spirellus*, *L.subdianicus* Tang, Yin, Peng, Ubick & Griswold, 2008, *L.subspirellus* and *L.torsivus* (Song et al. 1999, Tang et al. 2007, Tang et al. 2008, Liu et al. 2020c). Maybe it is a superficial perspective in previous work, but it still needs to be confirmed by future collection and further research.

## Supplementary Material

XML Treatment for Lysiteles nanfengmian
